# The Hypothalamus of the Beaked Whales: The Paraventricular, Supraoptic, and Suprachiasmatic Nuclei

**DOI:** 10.3390/biology12101319

**Published:** 2023-10-09

**Authors:** Simona Sacchini, Cristiano Bombardi, Manuel Arbelo, Pedro Herráez

**Affiliations:** 1 Veterinary Histology and Pathology, Atlantic Center for Cetacean Research, University Institute of Animal Health and Food Safety (IUSA), Veterinary School, University of Las Palmas de Gran Canaria, c/Transmontaña, s/n, 35416 Arucas, Spain; manuel.arbelo@ulpgc.es (M.A.); pedro.herraez@ulpgc.es (P.H.); 2 Department of Morphology, Campus Universitario de San Cristobal, University of Las Palmas de Gran Canaria, c/Blas Cabrera Felipe s/n, 35016 Las Palmas de Gran Canaria, Spain; 3 Department of Veterinary Medical Science, University of Bologna, Ozzano dell’Emilia, 40064 Bologna, Italy; cristiano.bombardi@unibo.it

**Keywords:** hypothalamus, beaked whales, toothed whales, supraoptic nucleus, paraventricular nucleus, suprachiasmatic nucleus

## Abstract

**Simple Summary:**

Beaked whales are singular and unconventional marine mammals, living in deep offshore waters. There is a scarce, almost absent, number of neuroanatomical studies on these toothed whales. The hypothalamus is a small brain region and it serves as the primary connection between the nervous and endocrine systems. This region is responsible for maintaining the body in a steady state of equilibrium known as homeostasis. The hypothalamic paraventricular and supraoptic nuclei of the Cuvier’s beaked whale and Blainville’s beaked whale are characterized here. A hypothalamic suprachiasmatic nucleus, the central biological clock, is also described for the first time in the two animals. The paraventricular nucleus occupied the preoptic region and the anterior or suprachiasmatic regions. The supraoptic nucleus was located in the preoptic, supraoptic, and tuberal regions. The suprachiasmatic nucleus was located in the ventromedial extremity of the tuberal hypothalamic region, occupying the median eminence of the hypothalamus. This study adds new insights and sets the stage for future investigations into the brains of beaked whales.

**Abstract:**

The hypothalamus is the body’s control coordinating center. It is responsible for maintaining the body’s homeostasis by directly influencing the autonomic nervous system or managing hormones. Beaked whales are the longest divers among cetaceans and their brains are rarely available for study. Complete hypothalamic samples from a female Cuvier’s beaked whale and a male Blainville’s beaked whale were processed to investigate the paraventricular (PVN) and supraoptic (SON) nuclei, using immunohistochemical staining against vasopressin. The PVN occupied the preoptic region, where it reached its maximum size, and then regressed in the anterior or suprachiasmatic region. The SON was located from the preoptic to the tuberal hypothalamic region, encompassing the optical structures. It was composed of a retrochiasmatic region (SONr), which bordered and infiltrated the optic tracts, and a principal region (SONp), positioned more medially and dorsally. A third vasopressin-positive nucleus was also detected, i.e., the suprachiasmatic nucleus (SCN), which marked the end of the SON. This is the first description of the aforementioned nuclei in beaked whales—and in any marine mammals—as well as their rostro-caudal extent and immunoreactivity. Moreover, the SCN has been recognized for the first time in any marine mammal species.

## 1. Introduction

The hypothalamus is responsible for maintaining the body’s homeostasis. Despite being relatively small, this diencephalic region is crucial for maintaining homeostasis and houses the complete system for basic life support. In fact, one of its main functions is to control the release of hormones from the pituitary gland. Several nuclei are present in this little region of the brain, which can be subdivided into three mediolateral compartments (lateral, medial, and periventricular) and four anteroposterior compartments (preoptic, anterior, tuberal, and mammillary) [1,2]. The hypophyseal stalk, which contains the hypothalamo-hypophyseal tract projecting mostly from the magnocellular neurons populating the paraventricular (PVN) and supraoptic (SON) nuclei to the neurohypophysis, connects the hypothalamus to the hypophysis. These neurons release oxytocin and vasopressin, which are then stored in the neurohypophysis. A grayish protrusion, known as the tuber cinereum (cinereus means gray), is caused by the infundibulum at the hypothalamic base [3]. Thus, the hypothalamo-neurohypophysial system, the archetypal instance of a neuroendocrine system, is formed by the SON and PVN, and their axons travel to the neurohypophysis. The portal capillaries in the PVN, which transport peptides to the anterior pituitary, are home to a second class of neuroendocrine neurons. Peptides from a third class of PVN neurons operate as neurotransmitters and neuromodulators when they project to other neurons [4]. The neurons of the parvicellular portion of the PVN contain a variety of neuroactive peptides, some of which are present alone or in combination with others [5]. Parvicellular neurons of the PVN give rise to descending axons that reach the brainstem and the spinal cord [6]. As a matter of fact, the hypothalamus is also part of the hypothalamic–pituitary–adrenal axis, which is a key system in the body’s reaction to stress. The axis leads to the synthesis of cortisol from the adrenal glands and the release of the corticotropin-releasing factor/hormone (CRF/CRH), produced primarily by the parvicellular population of the PVN. The CRF influences numerous areas of the brain; as a result, it modulates stress responses in the brainstem and neocortex [7].

In mammals, the suprachiasmatic nucleus (SCN) is a bilateral paired structure immediately above the optic chiasm. SCN is the principal circadian pacemaker in the mammalian brain. When the SCN is damaged, circadian rhythms of sleep–wakefulness and hormone release vanish [8]. In humans, the SCN degenerates in Alzheimer’s disease and type 2 diabetes variably alters the amount of its vasopressin-expressing neurons, harming the biological clock’s daily rhythmicity [9]. Along with the SCN, but with relative resistance to many forms of neurodegenerative pathology, PVN and SON may show neurodegenerative occurrences [10], even if, at first, the opposite was speculated [11].

### 1.1. The Hypothalamus of Toothed Whales: Very Little Data

Very little has been documented regarding the hypothalamus of marine mammals. In dolphins, the hypothalamic region is significantly foreshortened in the anterior–posterior diameter. The third ventricle’s floor and the posterior wall of the infundibulum are separated in the transverse plane from the interpeduncular space by a deep vertical cleft of the mesencephalic flexure. In gross and histological examinations, the mammillary bodies are difficult to distinguish [12]. Although not especially apparent, the anterior, tuberal, and posterior hypothalamus nuclei are visible [13]. Large hyperchromatic cells in the supraoptic and paraventricular nuclei, specifically well-formed in the latter, make them noticeable. The supraoptic commissure in dolphins is well-developed and organized, much like it is in other animals. The hippocampus, postcommissural fornix, and mammillothalamic tract are all correlated with the modest size of the mammillary bodies, which do not protrude at the brain’s surface in postnatal animals [14]. In the harbour porpoise (*Phocoena phocoena*, Linnaeus 1758, family *Phocoenidae*) four groups of orexin-A immunopositive neurons have also been described: the parvocellular, distributed in the medial zone of the hypothalamus mainly in a paraventricular region; the magnocellular, the main cluster identified throughout the hypothalamus, lateral to the parvocellular neurons; the optic tract cluster, extending well into the optic tract; and the zona incerta cluster, occupying the proximal part of the zona incerta [15].

### 1.2. On the Neuroanatomy of the Beaked Whales

To implement the current knowledge on the brain’s neuroanatomy in the beaked whales (BWs), we identify the brain regions involved in the activation of the hypothalamic–pituitary–adrenal axis, namely the PVN and the SON, establishing their neuroanatomical references by using histochemistry and immunohistochemistry. BWs are a cryptic family of toothed whales (TWs) with a stereotypic diving pattern consisting of single deep foraging dives—on average deeper than 1000 m—succeeded by a series of short, relatively shallow dives, aimed at avoiding predators like sharks and/or killer whales (*Orcinus orca*) [16]. BW strandings have been related to loud noise events linked to military exercises across the world [17].

Fresh samples of BW brains are really exclusive. In fact, few details about their neuroanatomy are known. Existent biometric and morphometric brain data of the two examined BWs are very scarce. We have one source for the Cuvier’s BW [18,19]; its brain weight and body weight (g) were 2004/2,273,000. In the case of the Blainville’s BW, one study provided data from three specimens, with a mean brain weight/body weight (g) of 1463/767,000 [20]; in a second study, three other specimens were examined, with their brain mass recorded as 1240 g [19].

Very few works include neurohistological data on the BW brain, including the cytoarchitecture of the visual cortex shown in two specimens of Cuvier’s BW [21], and the primary visual and primary auditory cortex revealed in one specimen of Cuvier’s BW [22]. Other sources provide some data on cortical thickness, cerebellum mass, cortex surface area, or neuron density values, mainly in the Cuvier’s BW [19,21,23]. Extrapolated data indicate that BWs may be able to dive deeper and for longer periods of time than dolphins because they have smaller brains, cerebella, and a lower number of cortical neurons [23].

## 2. Materials and Methods

For the study of the hypothalamus, five brains were obtained as follows: Two complete hypothalamic samples were from one adult female Cuvier’s beaked whale (*Ziphius cavirostris*, Cuvier 1823, family *Ziphiidae*) and one adult male Blainville’s beaked whale (*Mesoplodon densirostris*, De Blainville 1817, family *Ziphiidae*). Additionally, two striped dolphins (*Stenella coeruleoalba*, Meyen 1833, family *Delphinidae*), one Atlantic spotted dolphin (*Stenella frontalis*, (Cuvier 1829, family *Delphinidae*), and one common dolphin (*Delphinus delphis*, Linnaeus 1758, family *Delphinidae*) provided partial samples of the hypothalamus and/or were used for macroscopical images. Brains were removed and immersion-fixed at the time of necropsy in 4% formaldehyde in phosphate-buffered saline (PBS; pH 7.4). A detailed protocol on brain handling and sampling, as well as histochemical and immunohistochemical procedures, is available from a previously published work [24]. Brains were then sectioned in transverse (cross-) sections. The hypothalamus was cut rostrocaudally in a sequential form, using a sliding freezing microtome. In order to find common references in each hypothalamus, nine sections were selected as main references and were as equidistant as possible. The first series of sections adjacent to immunolabeled sections were stained for thionine (Lauth’s violet) to identify the cytoarchitectonic boundaries of the nuclei, discard neuropathological changes, and check the quality of the tissues [24]. Immunoperoxidase staining was carried out on free-floating 50 μm-thick coronal sections as follows. To block non-specific binding, sections were enhanced in a solution containing 10% normal serum and 0.5% Triton X-100 (Merck, Darmstadt, Germany) to permeabilize the tissue; this was conducted in PBS for 2 h at room temperature (RT). Thereafter, sections were incubated in the primary antibody overnight at 4 °C (Table 1).

The next day, sections were treated with a secondary antibody for 45 min at RT in a solution containing 1% normal serum in PBS after being rinsed in PBS (3 × 10 min) (Table 1). Following rinsing in PBS, the sections were incubated for one hour at RT with an avidin–biotin complex (ABC, Vector Laboratories (Newark, CA, USA); PK-4000). A 3,3′-diaminobenzidine (DAB) peroxidase kit (Vector Laboratories, SK-4100) was then used to react the sections. Finally, after mounting the sections on coated slides, they dried overnight. After that, slides were cleaned in xylene, dehydrated in ethanol, and coverslipped with Entellan (Merck, Darmstadt, Germany). The neuronal morphometric study was held by photographing neurons at 40× magnifications, using the Olympus XC50 digital camera. The measures were taken employing software for digital images (CellSens Standard, Olympus, Tokyo, Japan). Neurons with well-defined/no blurred borders and with a visible nucleolus were selected and measured. The shapes and sizes of the neurons and the number of dendrites were used as indicators to classify neurons and their populations.

### Specificity of the Antibodies

The primary antibodies were tested in different species of cetaceans, and positive controls were anteriorly carried out in a previous investigation concerning the methodology and neuromarkers for cetacean brains [24]. Vasopressin immunopositivity was extremely specific for the hypothalamic PVN, SON, and SCN. For this reason, it was chosen as the neuromarker of reference for the characterization of these nuclei.

The amino acid sequence of the cetacean vasopressin has only been studied in the fin whale (*Balaenoptera physalus*, Linnaeus 1758, family *Balaenopteridae*). Vasopressin was characterized through chemical and enzymatic methods, revealing that it is the same as in most other mammals but the difference resides in the proportion, with vasopressin being about 5 times more abundant than oxytocin [25,26]. In addition, the corticotropins (ACTH or adrenocorticotropic hormone) from two species of whales, sei whale (*Balaenoptera borealis*, Linnaeus 1758, family *Balaenopteridae*), and fin whale were found to be structurally identical to human corticotropin [27].

No studies on CRF are available but the Ensembl genome browser (www.ensembl.org, accessed on 23 September 2023) shares comparative genomics and sequence variations.

The percentage of the bottlenose dolphin (*Tursiops truncatus*, Montagu 1821, family *Delphinidae*) CRF orthologous sequence matching the human sequence is over 85%. The whole genome alignment coverage is 100. The amino acid sequence of the dolphin CRF is 193, while it is 196 in humans. The CRF orthologous sequence of the blue whale (*Balaenoptera musculus*, Linnaeus, 1758, family *Balaenopteridae*) matches the dolphin sequence by over 97%, having an amino acid sequence of 193, similar to the dolphin.

The percentage of the beluga whale (*Delphinapterus leucas*, Pallas 1776, family *Monodontidae*) and sperm whale (*Physeter macrocephalus*, Linnaeus 1758, family *Physeteridae*) ADH orthologous sequences matching the human sequence is over 87%.

The whole genome alignment coverage is 100. The ADH amino acid sequence for both animals is 166, while in humans, it is 164.

We can, therefore, assume that the amino acidic sequence of the ADH and CRF is retained along the order Cetacea and among its different families.

Finally, by substituting the primary antiserum with PBS, the staining was eliminated, which allowed for a final test of the secondary antibody’s specificity.

## 3. Results

### 3.1. The Hypothalamus of the Toothed Whales

The hypothalamic region of the examined species was well-developed despite its small size in comparison to the total volume of the brain. It was situated close to the third ventricle, caudal to the anterior commissure and the striatum, rostral to the midbrain, dorsal to the optic tract, the tuber cinereum, and the mammillary bodies, and ventromedial to the thalamus. The transition between the hypothalamus and the mesencephalon coincided with the appearance of the elliptic nucleus.

**Figure 1:**
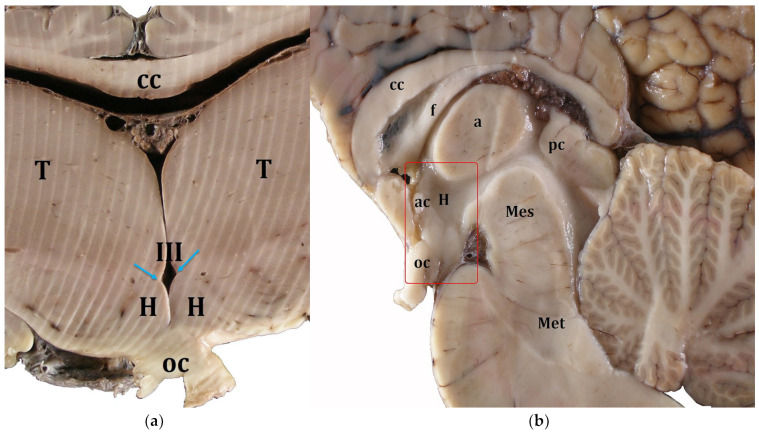
Gross anatomy of the hypothalamus and the adjoining structures. Cuvier’s beaked whale, transverse plane (**a**) and Atlantic spotted dolphin, midsagittal plane (**b**). Red rectangle in (**b**) indicates the hypothalamic region. a, Interthalamic adhesion; ac, anterior commissure; cc, corpus callosum; f, fornix; H, Hypothalamus; Mes, mesencephalon; Met, metencephalon; oc, optic chiasm; pc, posterior commissure; T, Thalamus; III, third ventricle; blue arrows, hypothalamic sulci.

In the cross-section, the hypothalamus (H) was located medially and ventrally to the thalamus (T) and dorsally to the optic chiasm (oc) (Figure 1a). The hypothalamic sulcus separated H from T above (Figure 1a, blue arrows).

In the midsagittal section (Figure 1b), H had a rhomboid shape and was located ventrally to the thalamus, specifically ventral to the interthalamic adhesion (a), dorsal to the optic chiasm (oc), caudal to the anterior commissure (ac), and rostral to the mesencephalon or midbrain (Mes) (Figure 1b). No evident round-shape mammillary bodies were recognizable in any of the examined species.

In the horizontal section, made at the level of the amygdaloid complex, the hypothalamus presented a rectangular shape, located around the third ventricle, medial to the thalamus, caudal to the anterior commissure, and rostral to the red and elliptical nuclei of the midbrain.

**Figure 2:**
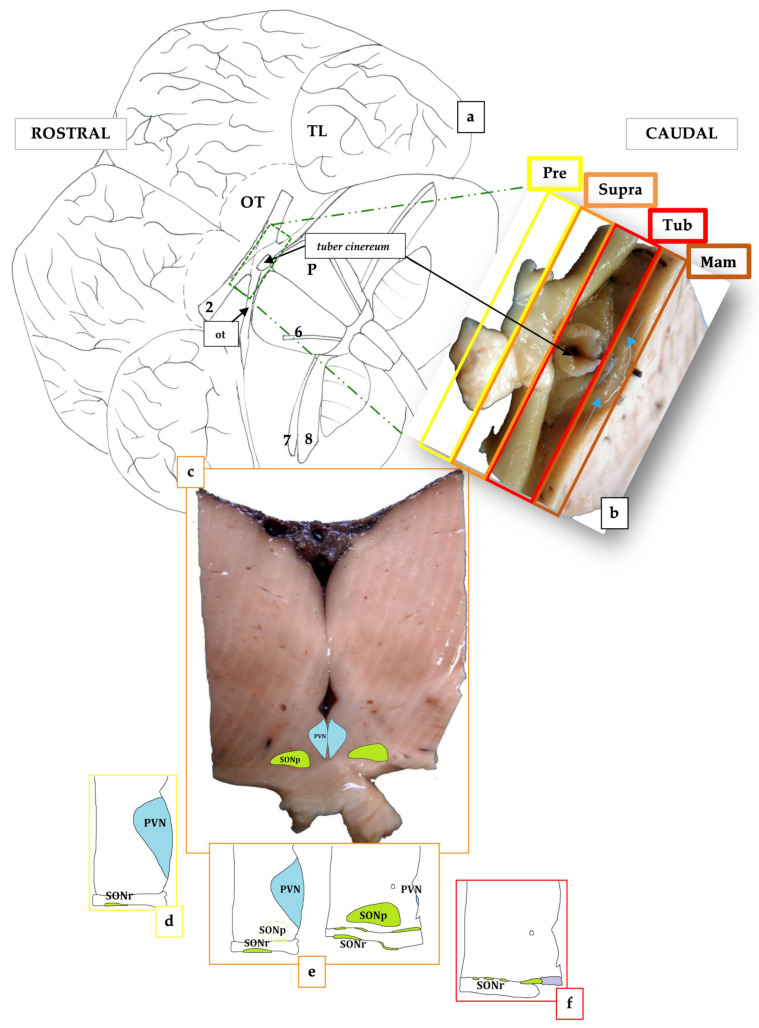
Scheme of the basal surface of the BW brain (**a**). Sampling from the same brain shown in Figure 1a; Blainville’s beaked whale (**b**,**c**). Subdivisions of the hypothalamic regions (ventral view); the 4 regions of the hypothalamus are: preoptic (Pre), suprachiasmatic (Supra), tuberal (Tu), and mammillary (Mam). The blue arrows indicate the rudimentary mammillary bodies (**b**). Anterior view of figure (**b**) showing the supraoptic area with PVN and SON (**c**). Plots of the preoptic (**d**), supraoptic (**e**), and tuberal (**f**) left regions of the BWs brain (**d**–**f**). In (**f**) the purple area corresponds to the SCN. 2, optic nerve; 6, abducens nerve; 7, facial nerve; 8, vestibulocochlear nerve; OT, olfactory tubercle; ot, optic tract; P, pons; TL, temporal lobe.

Examining the brain on its ventral face, it was possible to subdivide the hypothalamus into four regions according to the structures present ventrally to it (Figure 2a,b):

- Preoptic (Pre), rostral to the optic chiasm;
- Supraoptic or anterior (Supra), dorsal to the optic chiasm;
- Tuberal (Tub), dorsal to the tuber cinereum;
- Mammillary (Mam), dorsal to the rudimentary mammillary bodies.

The hypothalamus was continuous with the pituitary gland via the infundibulum. In the analyzed samples, the structures of the PVN and SON were well-defined. The PVN occupied the preoptic region and the anterior or suprachiasmatic region. The SON, the more extensive of the two, was located in the preoptic, supraoptic, and tuberal regions. The formation of the suprachiasmatic nucleus (SCN) marked the end of the SON. Using immunohistochemical staining against vasopressin (ADH), it was possible to specifically mark the neurons belonging to the PVN and the SON.

### 3.2. The Paraventricular Nucleus of BWs

**Figure 3:**
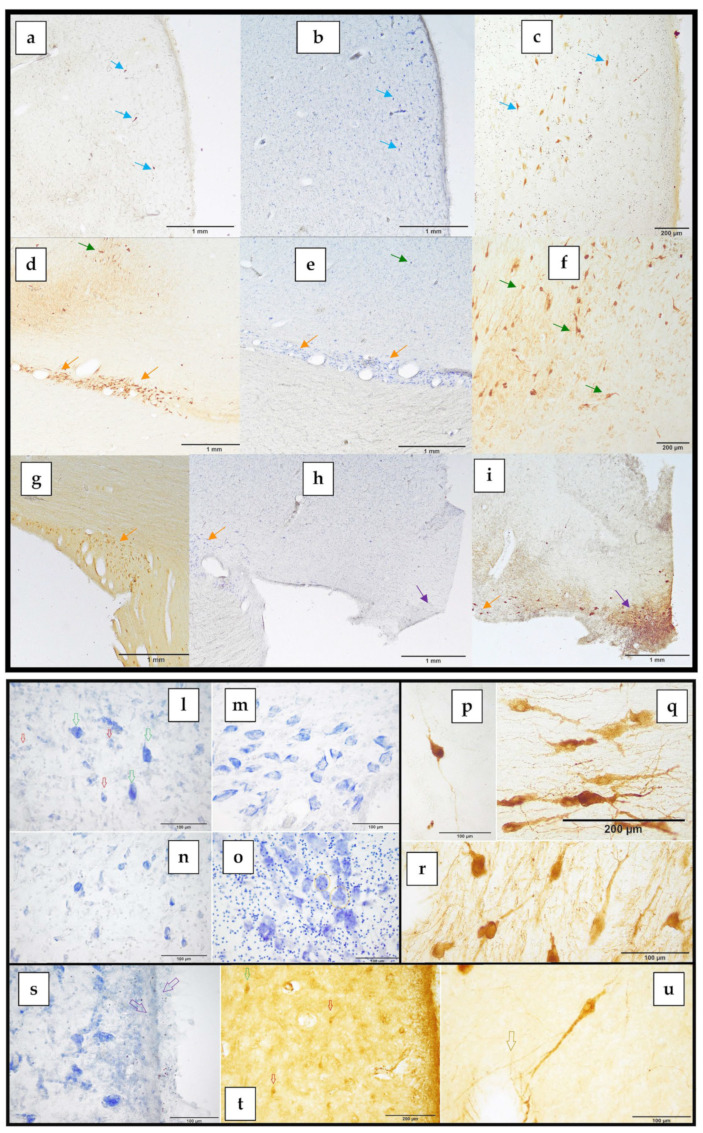
Microphotographs of Nissl and immunolabeled brain section of the PVN (**a**–**c**, blue arrows), SONr (**d**,**e**,**g**–**i**, orange arrows), SONp (**d**–**f**, green arrows), and SCN (**h**,**i**, purple arrows). Blainville’s BW: (**a**,**b**,**d**–**f**,**h**,**i**). Cuvier’s BW: (**c**,**g**). Blainville’s BW is more represented due to the higher quality of its tissues and subsequently, the immunolabeling. (**b**,**e**), and (**h**) are Nissl stained. Higher magnification of PVN and SON. Magnocellular (green arrows) and parvocellular (red arrows) neurons of the PVN; the polygonal morphology of both populations and the larger size of the magnocellular population are observed (**l**). Magnocellular neurons of polygonal morphology of the SONr (**m**) and SONp (**n**) regions. The big cells of the magnocellular group, here observed in the PVN, but also in the SON, are morphologically comparable, with Nissl material dispersed peripherally and, sometimes, colloidal cytoplasmic inclusions associated with neurosecretion products (orange circles, **o**). Figures (**l**–**o**) were Nissl stained. Immunostaining of a PVN magnocellular neuron (**p**). Magnocellular “horizontal” neurons of SONr (**q**) and magnocellular “vertical” neurons of SONp (**r**). Histological figures between p and r were immunostained against ADH. Magnocellular neurons of polygonal morphology of the PVN and ependymal cells (purple arrows, **s**); Thionine staining. Mild neuronal immunoreactivity against CRF in the PVN; magnocellular (green arrows) and parvocellular (red arrows) neurons (**t**). Both SON and PVN are densely vascularized, and magnocellular neurons are often associated with blood vessels (orange arrow); ADH immunostaining (**u**).

The PVN was positioned in the paraventricular region of the hypothalamus, on the lateral walls of the third ventricle (Figure 2c–e and Figure 3a–c). It covered the preoptic and supraoptic regions (Figure 2c–e). Furthermore, it was situated dorsally and medially to the SON in all its extension (Figure 2d,e). The superior border of the nucleus was established by the hypothalamic sulcus, which also represented the limit between the thalamus and the hypothalamus. It significantly expanded caudally, increasing its cell density and changing its form from flat to triangular (Figure 2d,e). Its highly clustered neurons were near the third ventricle’s surface, immediately below the ependyma (Figure 3a–c,s). The PVN reached its maximum growth in the preoptic region, extending more laterally and acquiring, on each side of the hypothalamus, a scalene triangle shape, with the base resting on the ependyma of the third ventricle and the apex directed laterally (Figure 2d). At this level, the most proximal portion of the optic nerve could be observed, although it was difficult to maintain its weak union with the brain parenchyma. As the nucleus reached the suprachiasmatic or anterior hypothalamus area, the neuron count gradually began to decline. The most caudal portion of this nucleus ceased to have a triangular shape, acquiring a flattened morphology in close contact with the ventricular surface (Figure 2e). However, some ADH-immunopositive neurons could be observed, positioned near the wall of the third ventricle, and progressively disappeared.

### 3.3. The Supraoptic Nucleus of BWs

The SON extended from the preoptic to the tuberal region (Figure 2c–f). It bounded all the optical way structures (the proximal portion of the optic nerve, optic chiasm, and distal optic tract) with its neurons and neuronal processes. The first neurons of the SON, formed the retrochiasmatic portion of the supraoptic nucleus (SONr), which appeared in the preoptic area, infiltrating the optic nerve (Figure 2d,e). In fact, the SON was divided into a retrochiasmatic region, which extended laterally and also bordered the optic tract, and a principal region (SONp), more medially and dorsally to the anterior (Figure 2e). The SONr region was the larger of the two divisions, being the first to emerge and continuing when the SONp became absent, caudally. The SONr region was the first to emerge and was the most extensive with respect to SONp and PVN, reaching the tuberal area (Figure 2f). In the supraoptic region, a notable increase in the size and neuronal density of the SONp region could be observed, which coincided with the regression of the PVN (Figure 2e). Hence, the SONp acquired a teardrop shape with the base facing the third ventricle. In addition, numerous immunoreactive ADH neurons were observed both at the level of the SONp region and at the level of the SONr, even infiltrating the optic tract (Figure 3d,e,g,h). The SONp reached its greatest extension in the supraoptic region (Figure 3f). However, at the end of the supraoptic region, the SONp disappeared, leaving only a few neurons. Entering the tuberal region, and in accordance with the optic tract involution, the SONr region, the only portion that remained of the two analyzed nuclei, also reduced in size (Figure 2f). At the same time, another hypothalamic nucleus appeared, also identified by its marked immunoreactivity against vasopressin: the SCN, located at the ventromedial extremity of the tuberal hypothalamus (Figure 3h,i). As the SCN grew in size, mainly laterally, the SONr progressively reduced its extent.

### 3.4. The Suprachiasmatic Nucleus of BWs

The SCN was not initially included in the present investigation. Despite that, the nucleus was evident and stained intensely against ADH. It was located in the ventromedial tuberal hypothalamic region, adjacent to the optic recess of the third ventricle and, thus, occupied the median eminence of the hypothalamus (Figure 2f and Figure 3h,i). Its neurons showed a very irregular morphology and an intense positivity to ADH, both at the level of the neuronal body and along its dendrites. This nucleus marked the end of the SON.

### 3.5. Cytoarchitecture of the PVN and SON

The neurons observed in the PVN and SON belonged to two cell populations:

1. The magnocellular neurons present in both the PVN and the SON;
2. The parvocellular neurons present exclusively in the PVN.

In Table 2, the mean area and perimeter ± standard deviation of the magnocellular and parvocellular neurons are shown. In addition, measurements performed in the thionine-stained magnocellular neurons in the 2 BWs are also displayed. In Cuvier’s BW (female) the perimeters and area of the magnocellular neurons are bigger than in the Blainville BW (male).

ADH primarily marked the magnocellular population. The immunoreaction was intense, allowing us to clearly visualize both the PVN and the SON and their total dimensions. For this reason, ADH was used as the cellular neuromarker of choice for these two hypothalamic nuclei. ADH immunoreactivity was strongly visible in the magnocellular population, both in the PVN and the SON. The PVN was made up of two cell populations: the magnocellular population made up of uniformly large neurons and the parvocellular population made up of smaller neurons (Figure 3l, green and red arrows, respectively). The SON was composed of a magnocellular population of large-sized neurons (Figure 3m,n). The large thionine-stained cells of the magnocellular group were morphologically similar, with the Nissl substance distributed peripherally. These neurons were large and polygonal in shape, with 3 to 5 sides (Figure 3l–n). The Nissl or tigroid substance was distributed in two ways: occupying the central zone or, as mostly observed in the SON, limiting itself to the periphery of the neurons (Figure 3m). The large nucleus with an evident nucleolus was located in a centric or slightly eccentric position, in most cases displacing the Nissl substance toward the periphery of the neuronal soma (Figure 3m). Colloidal cytoplasmic inclusions compatible with neurosecretory products were occasionally observed (Figure 3o, orange circles). ADH immunohistochemistry staining revealed the presence of PVN neurons, polygonal in shape, with 3 to 5 sides, and stained intensely both at the soma and dendrites (Figure 3p). However, some neurons stained more intensely than others (Figure 3c). SON had large, intensely stained perikaryal, both in its retrochiasmatic (Figure 3q) and principal (Figure 3r) divisions, occupying the basal surface of the hypothalamus and the optic tract. The orientation of the neurons was horizontal in the SONr region and vertical in the SONp (Figure 3q,r). Sometimes, it was difficult to differentiate the boundaries between the PVN and the SONp. Despite this, a more detailed observation of the distribution of the neurons allowed to establish the demarcation of the SONp region, since in this region the neurons were characterized by having a vertical and less dense distribution with respect to the PVN. This difference became more evident through the immunostaining against ADH and, as previously mentioned, in the neurons of the SONp, two dendrites stood out, one dorsal and the other ventral, arranged at opposite poles (Figure 3r). SONp had more dispersed neurons and its neuropil determined a fine and exuberant network of neuronal dendrites, infiltrating the optic chiasm. SONr was characterized by elongated, polygonal neurons, oriented parallel to the optic tract and chiasm or perpendicular when infiltrating the optical structures (Figure 3d,e,g). In the SONr, neurons were more numerous, and densely aggregated, and both the axon cones and long primary dendrites were intensely marked, which branched more distally into two secondaries (Figure 3q). In the PVN, the ependymal cells were identifiable as a single layer of cuboidal or columnar cells with microvilli and cilia, lying on the internal glial limiting membrane (Figure 3s). The parvocellular neurons of the PVN expressed a mild cytoplasmic positivity to CRF (Figure 3t). Immunostaining against CRF offered moderate labeling in the form of a finely granular immunoreaction, especially at the level of the parvicellular neurons in the PVN and, to a lesser extent, in the magnocellular neurons of PVN and SON. The positivity, when present, affected the soma and, to a lesser extent, the emergence cone of the dendrites. Magnocellular neurons, both in SON and PVN, were often associated with blood vessels (Figure 3u).

## 4. Discussion

The organization seen in other mammals, including some cetaceans like the common dolphin, was visible in the hypothalamus of the two BWs under study [28,29,30]. Similar to humans and other mammals, the hypothalamus was medial and ventral to the thalamus, ventral to the corpus callosum and the posterior commissure, dorsal to the optic chiasm, caudal to the main components of the striatum, and rostral to the midbrain [31,32]. Moreover, it displayed the normal subdivision found in other mammals, such as rats and humans [1,31,32]. Indeed, observing the brain on its ventral surface, it was possible to subdivide the hypothalamus into four areas: preoptic, supraoptic, tuberal, and mammillary dorsal to the rudimentary mammillary bodies. The term “mammillary” was coined in 1799 by Christian Friedrich Ludwig due to the breast appearance of these spherical structures of the brain [33]. This does not apply to the tiny cetaceans’ mammillary bodies. They lack the postnatal animal’s characteristic spherical shape protruding at the ventral surface of the brain, which concurs with the limited development of the hippocampus [14]. The supraoptic commissure in the analyzed BWs was well developed and structured like in humans. However, the floor of the BWs hypothalamus shortened as a result of the telescoping process, so that the interpeduncular transverse fossa, between the optic chiasm and the pons, had a slit shape, as previously described in the bottlenose dolphin [13]. This fact could justify a different arrangement in the BW PVN and SON. The fundamental feature of the analyzed species’ PVN is that it stayed in the preoptic area until it reached its maximal development, and then it involuted in the anterior or suprachiasmatic region. In humans, both the PVN and SON are localized in the suprachiasmatic and preoptic regions, respectively [32,34]. In contrast to the PVN, the SON had a significantly bigger expansion, growing from the preoptic hypothalamic area to the tuberal hypothalamic area, with a much more significant rostro-caudal development as compared to other mammals, such as humans [35]. As a result, SON infiltrated the full length of the optic structures (the proximal portion of the optic nerve, the optic chiasm, and the distal portion of the optic tract) with its neurons and neuronal processes. The Nomina Anatomica Veterinaria [36] recognizes two main regions of the SON: the pars suprachiasmatica and the pars postchiasmatica. However, there is some disagreement in the use of the terminology. In the rat brain, the use of three main regions prevails: a pars principalis with neurons attached to the lateral borders of the optic chiasm and optic tracts; a pars intraoptica, whose neurons are located among the optic axons of the optic chiasm, and the pars tuberalis, composed of neurons placed medially to the optic tracts [37]. In other mammals, like the Syrian or golden hamster (*Mesocricetus auratus*), the parcellation of the SON is limited to a retrochiasmatic and principal [38] or a medial and lateral [39] area. On the other hand, the Terminologia Neuroanatomica of the Federative International Programme for Anatomical Terminology (FIPAT) recognizes three main areas in humans (dorsolateral, dorsomedial, and ventromedial) [36], which are frequently validated in the published literature [34,35]. In the two BWs, the SON was divided into two different areas that we coined retrochiasmatic (SONr), which extended laterally and also surrounded the optic tract, and the principal (SONp), which was more medial and dorsal to the anterior. The SONr was the most extensive, in terms of its rostro-caudal development, being the first to appear and remaining when the SONp region disappeared caudally.

Regarding the PVN, the Nomina Anatomica Veterinaria registers a parvocellular paraventricular nucleus and a paired accessory nucleus, which is a neurosecretory group of neurons between the PVN and SON [40], while the Terminologia Neuroanatomica admits a paraventricular nucleus as a whole [36]. In our two BWs, the PVN was located in the periventricular region, and we identified two cell populations: the magnocellular population, with darkly stained big neurons, and the parvocellular population, with less intensely stained little neurons. SON was mostly populated by magnocellular neurons, which are easily detectable with Nissl staining. The hyperchromatic magnocellular neurons in the SON and PVN have been reported in other mammals, including humans [41,42], and were initially characterized by Gagel in 1928 [41].

Both the Nomina Anatomica Veterinaria and Terminologia Neuroanatomica agree with the existence of a suprachiasmatic nucleus with no subdivisions. This nucleus is widely recognized in mammals, including humans, but not in marine mammals, where it marked the end of the SON. A very remarkable particular of the two BWs is that SCN has been detected in conjunction with the very caudal part of the SON, in the tuberal region. SCN is usually detected more rostrally in many species like humans, rats, and even in flat-faced fruit-eating bats (*Artibeus planirostris*) [34,43,44]. Direct retinal projections to the SCN in adult mammals are in charge of resetting and syncing physiological and behavioral rhythms to light and dark cycles. In addition, different works have shown that laboratory rodents might suffer from a range of negative health and welfare effects that are linked to lighting settings that are frequently utilized in animal facilities [45]. On the basis of what was previously explained, the SCN in BWs could have a smaller volume and extent than other mammals, including other cetaceans. We do not exclude the possibility of a reduced extent of the SCN, even though blind animals, such as Kobe moles (*Mogera kobeae*), present normally developed SCN and retinohypothalamic projections. On the other hand, the pineal gland is the target of the primary polysynaptic efferents of SCN neurons [46]. However, most of the studies held on different cetaceans species agree that they lack the pineal gland. Noteworthy, a comprehensive study on 29 brains of the bottlenose dolphin species confirmed that this dolphin lacks a pineal gland and revealed extrapineal melatonin production by the ganglion cells in the retina, the Harderian gland, and the gut. In this scenario, the formation of a typical diurnal cycle may be hampered by recurrent variations in light intensity and spectral alterations in animals diving in low-light conditions [47]. Another nucleus usually described in the mediobasal part of the infundibular region is the arcuate or infundibular nucleus. The arcuate nucleus holds pivotal importance for the primary sensing of adiposity signals, such as leptin and insulin, and circulating nutrients, such as glucose [48]. Moreover, vasopressin and oxytocin are not detected in this nucleus [49], confirming the SCN as the detected nucleus. In fact, ADH is produced in the SCN in addition to the SON and PVN, while some ADH neurons can also be detected in the diagonal band of Broca, the nucleus basalis of Meynert, the bed nucleus of the stria terminalis [4], and the medial amygdala, which projects back to the PVN [50].

In humans, there are sex variations in the size of SCN vasopressin neurons and, thus, in their function, which are age-dependent [35]. We detected a sex variation in the magnocellular neurons, as in the female Cuvier’s BW, the perimeters and areas of the magnocellular neurons were bigger than in the male Blainville’s BW. Nevertheless, no sex differences have been detected in the human PVN and SON [11], and demonstrative measurements should be taken at different ages in the same species and sex.

## 5. Limitations of the Study

BWs are singular and unconventional animals to study, inhabiting deep, offshore waters. Their neuroanatomy is even more unusual to explore, evidenced by the scarce—almost absent—number of neuroanatomical studies, which exclusively concern the cerebral cortex. This attribute justifies the limited number of animals employed in this work. Additionally, the challenging peculiarity of investigating the BWs’ hypothalamus—as well as of any large-sized cetacean—lies in maintaining its integrity during brain extraction. The very delicate hypothalamic region is prone to rupture during dissection and extraction, especially around the optic chiasm, predominantly damaging the SON, the SCN, and the tuber cinereum. Compounding these challenges, post-mortem fixation times negatively affect the quality of brain tissues, and free-floating immunohistochemical procedures represent a very delicate moment where we can even lose some sections.

## 6. Conclusions

This is the first description of the PVN, SON, and SCN in beaked whales, as well as an exploration of their rostro-caudal development and immunoreactivity. SCN has been recognized for the first time in any marine mammal species, and it emerges as an important area for neuropathological sampling, as it has been recently shown that BWs are prone to developing neurodegenerative hallmarks.

## Figures and Tables

**Table 1:** The table shows the two primary antibodies used as neuromarkers for PVN, SON, and SCN. Vasopressin, antidiuretic hormone, arginine vasopressin, or argipressin (ADH); corticotropin-releasing factor/hormone (CRF/CRH); polyclonal (P); goat (G); rabbit (R); normal serum (NS); secondary antibody (SA); biotinylated anti-goat IgG (BaG); biotinylated anti-rabbit IgG (BaR).

Primary Antibody	Clonality Host Species Isotype	Species Reactivity	Dilution	NS	SA
Anti-Vasopressin Abcam Ab39363	P R IgG	Reacts with: Rat, Pig	1/500	G (S-1000)	BaR (BA-1000)
Anti-CRF1/CRHR1 Abcam Ab59023	P G IgG	Reacts with: Human Predicted to work with: Rat, Sheep, Chicken, Pufferfish, Rhesus monkey, African green	1/100	R (S-5000)	BaG (BA-9500)

**Table 2:** Mean area and perimeter ± standard deviation (SD) of the magnocellular and parvocellular neurons; par, parvocellular; mag, magnocellular; P, perimeter; A, area; ADH, vasopressin; CBW, Cuvier’s BW; BBW, Blainville’s BW.

	PVNpar	PVNmag	SONmag
Thionine P	49.23 ± 7.19 µm	91.23 ± 15.19 µm	87.05 ± 15.47 µm
Thionine A	139.16 ± 29.66 µm^2^	496.65 ± 155.4 µm^2^	445.11 ± 142.81 µm^2^
ADH P	-	100.77 ± 16.74 µm	100.37 ± 17.82 µm
ADH A	-	508.78 ± 149.61 µm^2^	558.21 ± 176.48 µm^2^
Thionine P CBW	-	96.87 ± 14.79 µm	89.02 ± 13.75 µm
Thionine A CBW	-	532.72 ± 165.41 µm^2^	485.98 ± 128.94 µm^2^
Thionine P BBW	-	87.48 ± 14.45 µm	86.25 ± 16.28 µm
Thionine A BBW	-	472.6 ± 145.75 µm^2^	423.28 ± 128.94 µm^2^

## Data Availability

The data presented in this study are available from the corresponding author upon reasonable request.

## References

[1] Paxinos G. (2004). The Rat Nervous System.

[2] Schröder H., Moser N., Huggenberger S., Schröder H., Moser N., Huggenberger S. (2020). The Mouse Hypothalamus. Neuroanatomy of the Mouse: An Introduction.

[3] Dudás B. (2021). Part I—Morphology of the human hypothalamus: Anatomy, blood supply, nuclei and pathways. Atlas of the Human Hypothalamus.

[4] Swaab D.F. (2003). Chapter 8 Supraoptic and paraventricular nucleus (SON, PVN). Handbook of Clinical Neurology.

[5] Kupfermann I., Kandel E.R., Schwartz J.H., Jessell T.M. (1991). Hypothalamus and limbic system: Peptidergic neurons, homeostasis, and emotional behavior. Principles of the Neural Science.

[6] Saper C.B., Paxinos G. (1990). Hypothalamus. The Human Nervous System.

[7] Vuppaladhadiam L., Ehsan C., Akkati M., Bhargava A. (2020). Corticotropin-Releasing Factor Family: A Stress Hormone-Receptor System’s Emerging Role in Mediating Sex-Specific Signaling. Cells.

[8] Ibata Y., Okamura H., Tanaka M., Tamada Y., Hayashi S., Iijima N., Matsuda T., Munekawa K., Takamatsu T., Hisa Y. (1999). Functional morphology of the suprachiasmatic nucleus. Front. Neuroendocrinol..

[9] Hogenboom R., Kalsbeek M.J., Korpel N.L., de Goede P., Koenen M., Buijs R.M., Romijn J.A., Swaab D.F., Kalsbeek A., Yi C.-X. (2019). Loss of arginine vasopressin- and vasoactive intestinal polypeptide-containing neurons and glial cells in the suprachiasmatic nucleus of individuals with type 2 diabetes. Diabetologia.

[10] Stewart C.A., Finger E.C. (2021). Chapter 7—The supraoptic and paraventricular nuclei in healthy aging and neurodegeneration. Handbook of Clinical Neurology.

[11] Goudsmit E., Hofman M.A., Fliers E., Swaab D.F. (1990). The supraoptic and paraventricular nuclei of the human hypothalamus in relation to sex, age and Alzheimer’s disease. Neurobiol. Aging.

[12] Morgane P.J., Jacobs M.S., McFarland W.L. (1980). The anatomy of the brain of the bottlenose dolphin (*Tursiops truncatus*). Surface configurations of the telencephalon of the bottlenose dolphin with comparative anatomical observations in four other cetacean species. Brain Res. Bull..

[13] Oelschläger H.H.A., Oelschläger J.S., Perrin W.F., Würsig B., Thewissen J.G.M. (2009). Brain. Encyclopedia of Marine Mammals.

[14] Morgane P., Jacobs M., Harrison R.J. (1972). Comparative anatomy of the cetacean central nervous system. Functional Anatomy of Marine Mammals.

[15] Dell L.A., Patzke N., Bhagwandin A., Bux F., Fuxe K., Barber G., Siegel J.M., Manger P.R. (2012). Organization and number of orexinergic neurons in the hypothalamus of two species of Cetartiodactyla: A comparison of giraffe (*Giraffa camelopardalis*) and harbour porpoise (*Phocoena phocoena*). J. Chem. Neuroanat..

[16] Baird R.W., Webster D.L., Schorr G.S., McSweeney D.J., Barlow J. (2008). Diel variation in beaked whale diving behavior. Mar. Mammal Sci..

[17] Bernaldo de Quirós Y., Fernandez A., Baird R.W., Brownell R.L., Aguilar de Soto N., Allen D., Arbelo M., Arregui M., Costidis A., Fahlman A. (2019). Advances in research on the impacts of anti-submarine sonar on beaked whales. Proc. R. Soc. B Biol. Sci..

[18] Ridgway S.H., Brownson R.H. (1984). Relative brain sizes and cortical surface areas in odontocetes. Acta Zool. Fenn..

[19] Ridgway S., Carlin K., Van Alstyne K., Hanson A., Tarpley R. (2017). Comparison of Dolphins’ Body and Brain Measurements with Four Other Groups of Cetaceans Reveals Great Diversity. Brain Behav. Evol..

[20] Marino L. (1998). A Comparison of Encephalization between Odontocete Cetaceans and Anthropoid Primates. Brain Behav. Evol..

[21] Graïc J.-M., Peruffo A., Corain L., Finos L., Grisan E., Cozzi B. (2022). The primary visual cortex of Cetartiodactyls: Organization, cytoarchitectonics and comparison with perissodactyls and primates. Brain Struct. Funct..

[22] Hof P.R., Chanis R., Marino L. (2005). Cortical complexity in cetacean brains. Anat. Record. Part A.

[23] Ridgway S.H., Brownson R.H., Van Alstyne K.R., Hauser R.A. (2019). Higher neuron densities in the cerebral cortex and larger cerebellums may limit dive times of delphinids compared to deep-diving toothed whales. PLoS ONE.

[24] Sacchini S., Herráez P., Arbelo M., Espinosa de los Monteros A., Sierra E., Rivero M., Bombardi C., Fernández A. (2022). Methodology and Neuromarkers for Cetaceans’ Brains. Vet. Sci..

[25] Acher R., Chauvet J., Chauvet M.T. (1964). Isolation of Finback Whale Oxytocin and Vasopressin. Nature.

[26] Chauvet J., Chauvet M.T., Acher R. (1963). The neurohypophysial hormones of mammals: Isolation and characterization of oxytocin and vasopressin from whale (*Balaenoptera physalus* L.). Bull. Soc. Chim. Biol..

[27] Kawauchi H., Muramoto K., Ramachandran J. (1978). Isolation and primary structure of adrenocorticotropin from several species of whale. Int. J. Pept. Protein Res..

[28] Grünthal E. (1942). Über den Primatencharakter des Gehirns von Delphinus delphis. Eur. Neurol..

[29] Hatschek R., Schlesinger H. (1902). Der Hirnstamm des Delphins (*Delphinus delphis* L.). Arbeiten aus dem neurologischen Institute (Institut für Anatomie und Physiologie des Zentralnervensystems) an der Wiener Universität. IX.

[30] Jelgersma G. (1934). Das Gehirn der Wassersäugetiere: Eine Anatomische Untersuchung.

[31] Barone R.B.R. (2004). Anatomie Comparèe des Mammifëres Domestiques. Tome 6: Neurologie 1 Systëme Nerveux Central.

[32] Parent A., Carpenter M. (1996). Carpenter’s Human Neuroanatomy.

[33] Ludwig C.F. (1779). De cinerea cerebri substantia. Exercitationes Academicae.

[34] Hofman M.A., Goudsmit E., Purba J.S., Swaab D.F. (1990). Morphometric analysis of the supraoptic nucleus in the human brain. J. Anat..

[35] Ishunina T.A., Swaab D.F. (1999). Vasopressin and oxytocin neurons of the human supraoptic and paraventricular nucleus: Size changes in relation to age and sex. J. Clin. Endocrinol. Metab..

[36] Federative International Programme for Anatomical Terminology (FIPAT) (2017). Terminologia Neuroanatomica. International Neuroanatomical Terminology.

[37] Crespo D., Ramos J., Gonzalez C., Fernandez-Viadero C. (1990). The supraoptic nucleus: A morphological and quantitative study in control and hypophysectomised rats. J. Anat..

[38] Navarro A., Tolivia J., Alvarez-Uría M. (1994). Hamster supraoptic nucleus: Cytoarchitectural, morphometric, and three-dimensional reconstruction. Anat. Rec..

[39] Delville Y., Koh E.T., Ferris C.F. (1994). Sexual differences in the magnocellular vasopressinergic system in golden hamsters. Brain Res. Bull..

[40] International Committee on Veterinary Gross Anatomical Nomenclature ICVGAN (2017). Nomina Anatomica Veterinaria.

[41] Gagel O. (1928). Zur Topik und feineren Histologie der vegetativen Kerne des Zwischenhirns. Zeitschrift für Anatomie und Entwicklungsgeschichte.

[42] Møller M., Busch J.R., Jacobsen C., Lundemose S.B., Lynnerup N., Rath M.F., Banner J. (2018). The accessory magnocellular neurosecretory system of the rostral human hypothalamus. Cell Tissue Res..

[43] Saper C.B., Lowell B.B. (2014). The hypothalamus. Curr. Biol..

[44] Nelyane N.M.S., Marília A.S.B., Helder H.A.M., Melquisedec A.D.S., Lara L.S., Paulo L.A.G.M., Fernando V.L.L., Jeferson S.C., Ruthnaldo R.M.L., Judney C.C. (2018). The Suprachiasmatic Nucleus and the Intergeniculate Leaflet of the Flat-Faced Fruit-Eating Bat (*Artibeus planirostris*): Retinal Projections and Neurochemical Anatomy. Front. Neuroanat..

[45] González M.M.C. (2018). Dim Light at Night and Constant Darkness: Two Frequently Used Lighting Conditions That Jeopardize the Health and Well-being of Laboratory Rodents. Front. Neurol..

[46] Wu Y.-H., Swaab D.F. (2005). The human pineal gland and melatonin in aging and Alzheimer’s disease. J. Pineal Res..

[47] Cozzi B., Huggenberger S., Oelschläger H., Cozzi B., Huggenberger S., Oelschläger H. (2017). Chapter 6. Brain, spinal cord, and cranial nerves. The Anatomy of Dolphins. Insights into Body Structure and Function.

[48] Jais A., Brüning J.C. (2021). Arcuate Nucleus-Dependent Regulation of Metabolism—Pathways to Obesity and Diabetes Mellitus. Endocr. Rev..

[49] Zimmerman E.A., Robinson A.G. (1976). Hypothalamic neurons secreting vasopressin and neurophysin. Kidney Int..

[50] Pardo-Bellver C., Cadiz-Moretti B., Novejarque A., Martinez-Garcia F., Lanuza E. (2012). Differential efferent projections of the anterior, posteroventral, and posterodorsal subdivisions of the medial amygdala in mice. Front. Neuroanat..

